# Nationwide Survival Benefit after Implementation of First-Line Immunotherapy for Patients with Advanced NSCLC—Real World Efficacy

**DOI:** 10.3390/cancers13194846

**Published:** 2021-09-28

**Authors:** Mette T. Mouritzen, Andreas Carus, Morten Ladekarl, Peter Meldgaard, Anders W. M. Nielsen, Anna Livbjerg, Jacob W. Larsen, Halla Skuladottir, Charlotte Kristiansen, Kim Wedervang, Tine Schytte, Karin H. Hansen, Anne-Cathrine Østby, Malene S. Frank, Jakob Lauritsen, Jens B. Sørensen, Seppo W. Langer, Gitte F. Persson, Jon L. Andersen, Johanna M. C. Frary, Lars B. Drivsholm, Charles Vesteghem, Heidi S. Christensen, Birgitte Bjørnhart, Mette Pøhl

**Affiliations:** 1 Department of Oncology, Aalborg University Hospital, Hobrovej 18-22, 9000 Aalborg, Denmark; andreascarus@rn.dk (A.C.); morten.ladekarl@rn.dk (M.L.); 2 Clinical Cancer Research Center, Aalborg University Hospital, Sdr. Skovvej 15, 9000 Aalborg, Denmark; charles.vesteghem@rn.dk (C.V.); h.soegaard@rn.dk (H.S.C.); 3 Department of Clinical Medicine, Aalborg University, Sdr. Skovvej 15, 9000 Aalborg, Denmark; 4 Department of Oncology, Aarhus University Hospital, Palle Juul-Jensens Boulevard 99, 8200 Aarhus, Denmark; petemeld@rm.dk (P.M.); andernls@rm.dk (A.W.M.N.); ANLIVB@rm.dk (A.L.); 5 Department of Oncology, Region Hospital West Jutland, Gl. Landevej 61, 7400 Herning, Denmark; jacobweje@gmail.com (J.W.L.); hallskul@rm.dk (H.S.); 6 Department of Oncology, Vejle Hospital, University Hospital of Southern Denmark, Beriderbakken 4, 7100 Vejle, Denmark; Charlotte.Kristiansen@rsyd.dk; 7 Department of Oncology, Hospital Sønderjylland, Sydvang 1, 6400 Sønderborg, Denmark; Kim.Wedervang@rsyd.dk; 8 Department of Oncology, Odense University Hospital, Sdr. Boulevard 29, 5000 Odense, Denmark; tine.schytte@rsyd.dk (T.S.); karin.holmskov@rsyd.dk (K.H.H.); Birgitte.Bjornhart@rsyd.dk (B.B.); 9 Department of Clinical Research, University of Southern Denmark, Winsløwparken 19, 3rd, 5000 Odense, Denmark; 10 Odense Patient Data Explorative Network (OPEN), J. B. Winsløws Vej 9a, 5000 Odense, Denmark; 11 Department of Clinical Oncology and Palliative Care, Zealand University Hospital, Sygehusvej 10, 4000 Roskilde, Denmark; anboe@regionsjaelland.dk (A.-C.Ø.); malf@regionsjaelland.dk (M.S.F.); Jakob.Lauritsen@regionh.dk (J.L.); 12 Department of Clinical Medicine, University of Copenhagen, Blegdamsvej 3B, 2000 Copenhagen, Denmark; Jens.Benn.Soerensen@regionh.dk (J.B.S.); Seppo.Langer@regionh.dk (S.W.L.); gitte.persson@regionh.dk (G.F.P.); 13 Department of Oncology, Rigshospitalet, Blegdamsvej 9, 2100 Copenhagen, Denmark; mette.poehl@regionh.dk; 14 Department of Oncology, Copenhagen University Hospital, Herlev/Gentofte, Borgmester Ib Juuls Vej 1, 2730 Herlev, Denmark; jon.alexander.lykkegaard.andersen@regionh.dk; 15 Department of Oncology, North Zealand Hospital, Dyrehavevej 29, 3400 Hillerød, Denmark; johanna@familjenkos.se (J.M.C.F.); lars.bo.drivsholm@regionh.dk (L.B.D.); 16 Department of Hematology, Aalborg University Hospital, Hobrovej 18-22, 9000 Aalborg, Denmark

**Keywords:** real-world evidence, cancer immunotherapy, immune checkpoint inhibitors, anti-PD-1, first-line treatment, non-small cell lung cancer, advanced lung cancer, clinical prognostic factors, overall survival, Danish registry

## Abstract

**Simple Summary:**

The expected change in overall survival (OS) in patients with advanced non-small cell lung cancer (NSCLC) after the clinical implementation of immune checkpoint inhibitor therapy (ICI) has not been substantially investigated in large real-world cohorts outside randomized controlled trials (RCTs). In this nationwide study, we compared OS before and after the implementation of ICI and found that 3-year OS tripled from 6% to 18%. Patients receiving ICI had a lower OS than demonstrated in RCTs, except for patients with performance status (PS) 0. More than a fifth of the patients progressed early within the first six ICI cycles. Adverse prognostic factors were PS ≥ 1 and metastases to the bone and liver.

**Abstract:**

Background The selection of patients with non-small cell lung cancer (NSCLC) for immune checkpoint inhibitor (ICI) treatment remains challenging. This real-world study aimed to compare the overall survival (OS) before and after the implementation of ICIs, to identify OS prognostic factors, and to assess treatment data in first-line (1L) ICI-treated patients without epidermal growth factor receptor mutation or anaplastic lymphoma kinase translocation. Methods Data from the Danish NSCLC population initiated with 1L palliative antineoplastic treatment from 1 January 2013 to 1 October 2018, were extracted from the Danish Lung Cancer Registry (DLCR). Long-term survival and median OS pre- and post-approval of 1L ICI were compared. From electronic health records, additional clinical and treatment data were obtained for ICI-treated patients from 1 March 2017 to 1 October 2018. Results The OS was significantly improved in the DLCR post-approval cohort (*n* = 2055) compared to the pre-approval cohort (*n* = 1658). The 3-year OS rates were 18% (95% CI 15.6–20.0) and 6% (95% CI 5.1–7.4), respectively. On multivariable Cox regression, bone (HR = 1.63) and liver metastases (HR = 1.47), performance status (PS) 1 (HR = 1.86), and PS ≥ 2 (HR = 2.19) were significantly associated with poor OS in ICI-treated patients. Conclusion OS significantly improved in patients with advanced NSCLC after ICI implementation in Denmark. In ICI-treated patients, PS ≥ 1, and bone and liver metastases were associated with a worse prognosis.

## 1. Introduction

Lung cancer remains the leading cause of cancer-related death worldwide; in Denmark, lung cancer is one of the most common cancer types with an annual incidence of approximately 5000 cases [1]. Non-small cell lung cancer (NSCLC) accounts for more than 80% of the cases; most Danish patients present with stage IIIB–IV disease at diagnosis and have poor 5-year survival rates of 3% [2]. During the past 5 years, treatment with immune-checkpoint inhibitors (ICIs) has transformed the advanced NSCLC treatment landscape. Improved OS was observed in patients receiving ICIs in the second or later lines of treatment [3,4,5]. Furthermore, in the first-line (1L) randomized controlled trials (RCTs), KEYNOTE-024 and KEYNOTE-042, the median overall survival (mOS) improved to 26.3 and 20 months with ICIs compared to 14.2 and 12.2 months with chemotherapy (CTx), respectively [6,7,8]. These results led to the approval of 1L ICI treatment in Denmark on 1 February 2017. Programmed Death-Ligand 1 (PD-L1) is currently used as a predictive biomarker for ICI treatment. PD-L1 ≥ 50% is the cut-off for 1L ICI monotherapy based on RCTs that enrolled patients with different PD-L1 cut-offs [5]. However, the efficacy of ICIs in highly selected patients included in the RCTs may not be reproducible in patients treated in a routine clinical setting because of the impact of patient-, provider-, and system-related factors [9,10]. Therefore, real-world studies (RWS) on ICIs in consecutively treated patients have focused on patient-related factors (age, Eastern Cooperative Oncology Group (ECOG) performance status (PS) ≥ 2, and brain metastases) [11]. These studies indicate that patients aged > 70 years have an mOS comparable to that of younger patients [12]. In addition, patients with brain metastases have an mOS comparable to that of patients without brain metastases [13,14]. By contrast, PS ≥ 2 has been associated with significantly reduced mOS, independent of treatment line, and a systematic review demonstrated a pooled mOS hazard ratio (HR) of 2.72 compared to PS 0–1 [15,16]. RWS indicate significantly reduced response rates and impaired mOS in patients with bone metastases (BoM) compared to those without [17,18]. This suggests a reduced ICI effect in patients with BM; however, more data from RCTs and larger RWS are warranted. The expected change in overall survival (OS) in patients with advanced NSCLC after the clinical implementation of ICIs has only been sparsely investigated [19,20].

This nationwide RWS aimed to compare the OS before and after the implementation of 1L ICI in patients with advanced NSCLC without epidermal growth factor receptor (EGFR) or anaplastic lymphoma kinase (ALK) molecular alterations. Furthermore, the aim was to uncover prognostic factors for OS and report on treatment data in patients treated with 1L ICI.

## 2. Material and Methods

### 2.1. Patients

#### 2.1.1. Cohorts from the Danish Lung Cancer Registry (DLCR)

**Figure 1:**
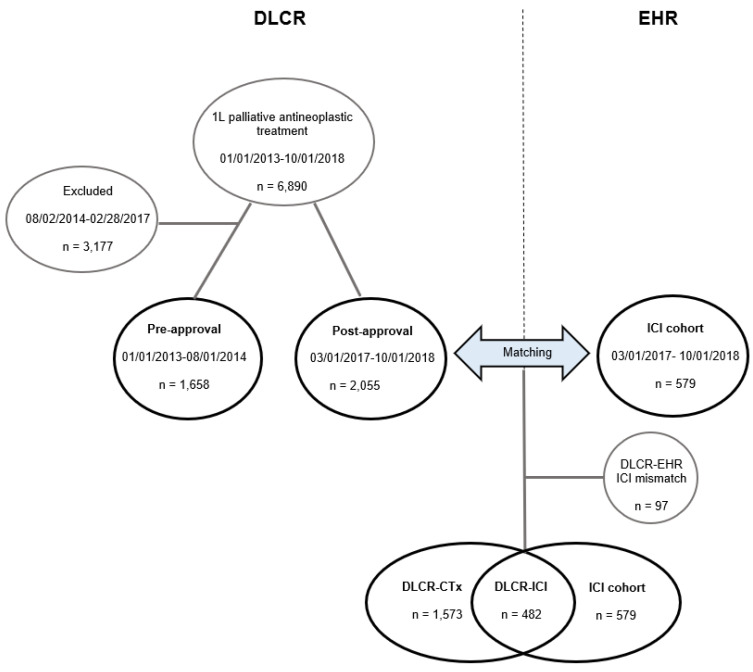
Flowchart showing the generation of the Danish Lung Cancer Registry (DLCR) cohorts before and after the approval of immune checkpoint inhibitors (ICIs). Treatment data from the electronic health records (EHRs) were applied on the DLCR post-approval cohort to divide patients into the DLCR-chemotherapy (CTx) and DLCR-ICI cohorts. Due to missing and inaccurate data in the DLCR, 97 ICI-treated patients identified from institutional records were not registered in the DLCR.

The DLCR, a part of the Danish Clinical Quality Program (National Clinical Registries), includes data automatically transferred from other national registries [21,22]. From the DLCR, baseline demographics and clinical data were extracted for patients with NSCLC, without EGFR/ALK molecular alterations, who started 1L palliative antineoplastic treatment from 1 March 2013 to 1 October 2018 (*n* = 6890) (Figure 1; Figure S1). This cohort was separated into a *DLCR pre-approval cohort*, comprising patients who started treatment before the approval of ICIs in any line (1 March 2013 to 1 August 2014; *n* = 1658), and a *DLCR post-approval cohort,* comprising patients who started treatment after the approval of 1L ICI in Denmark (1 March 2017 to 1 October 2018; *n* = 2055). To minimize the impact of second-line ICI (implemented in Denmark in September 2015), patients who started 1L treatment between 2 August 2014 and 28 February 2017 (*n* = 3177), were excluded (Figure 1).

#### 2.1.2. ICI Cohort Identified from Electronic Health Records (EHRs)

Data on PS and metastatic sites, and antineoplastic treatment details are lacking in the DLCR. To obtain these data on the 1L ICI-treated patients, the nationwide *ICI cohort* of consecutive patients initiating 1L ICI-treatment between 1 March 2017 and 1 October 2018 (*n* = 579) in all oncology departments administering ICIs in Denmark (*n* = 11) was identified. EHRs were reviewed in order to obtain clinical and treatment data on the ICI-treated patients.

#### 2.1.3. Matching of the DLCR Post-Approval Cohort and the EHR-Identified ICI Cohort

Stratification according to systemic antineoplastic treatment in the *DLCR post-approval cohort* was accomplished by matching with the EHR-identified *ICI cohort*. A match of 83% was observed, and the ICI-treated patients in the *DLCR post-approval cohort* were identified (*DLCR-ICI cohort*, *n* = 482). Thus, 97 patients identified from institutional records were not included in the *DLCR post-approval cohort* (*mismatch*; Figure 1). According to the national treatment guidelines at that time, the standard 1L treatment of the remaining patients in the DLCR post-approval cohort was platinum-doublet CTx (*DLCR-CTx cohort*; *n* = 1573) (Figure 1).

Hence, two different ICI cohorts were identified. The *DLCR-ICI cohort* that was used in the analyses comparing the OS before and after the implementation of 1L ICI, and the EHR-identified *ICI cohort* that was used in the detailed analyses of ICI-related clinical outcomes and treatment data.

### 2.2. Data Management of the EHR-Identified ICI Cohort

Due to our study definition of 1L treatment (first palliative treatment after NSCLC diagnosis or at relapse ≥ 6 months after curatively intended treatment), 12 patients (2%) received nivolumab (3 mg/kg every 2 weeks). ICI doses were prescribed according to Danish guidelines at the time, with a fixed pembrolizumab dose at 200 mg or 2 mg/kg every 3 weeks for a maximum of 2 years. Individual ICI dose intensities (mg/kg/time) were not recorded [23]. The reasons for ICI discontinuation were recorded, and the types of immune-related adverse events (irAEs) leading to ICI discontinuation were recorded. Additionally, hospitalization due to irAEs was recorded as a dichotomous variable (yes/no). Radiologic assessments according to the Response Evaluation Criteria in Solid Tumors were not consistently available. Therefore, the date of disease progression was defined as the date of radiologically-verified progressive disease (PD). If no radiological PD was evident, the date of PD was defined as the first clinical evidence of PD leading to ICI discontinuation. The index date was defined as the date of the first ICI administration. For patients still alive, the censoring date was 1 March 2020, and the date of last follow-up was defined as the last EHR-documented patient contact. Time-to-event measures were OS, progression-free survival (PFS), and time to treatment discontinuation (TTD).

### 2.3. Statistical Methods

#### 2.3.1. The DLCR Cohorts

The chi-square test was used to test for differences in categorical baseline characteristics between the pre- and post-approval cohorts, similarly to the DLCR-CTx and DLCR-ICI cohorts. The TNM stage was not considered due to the large proportion of missing values in the DLCR. Kaplan–Meier (KM) estimates were used to assess OS, and the log-rank test was used to compare the estimated survival curves.

#### 2.3.2. The EHR-Identified ICI Cohort

KM estimates were used to assess OS, PFS, and TTD, and log-rank tests were used to test for differences according to baseline characteristics. In the survival analyses, the Charlson Comorbidity Index Score (CCIS) was categorized as 0–1 and ≥2. Smoking status was excluded from the analyses due to a limited number of “never smokers” and the heterogenous smoking patterns in the “former smoking” group. TNM stage was excluded as a covariate from the survival analyses because of its interaction with metastatic sites. The remaining baseline characteristics were included as covariates and, for each of them, the assumption of proportional hazard function was assessed. Since the ECOG PS violated the assumption, weighted univariable and multivariable Cox regressions were used [24]. Multivariable Cox regression analysis was extended with an interaction between sex and histopathology. Survival analyses were not adjusted for age-related background mortality. The median follow-up was calculated using the reverse KM estimate.

All analyses were performed using R version 4.0.2 (R Core Team, Vienna, Austria) [25]. The survival- and ggsurvplot-packages were used to construct the KM estimates, and the coxphw package was used to perform the weighted Cox regressions.

## 3. Results

### 3.1. The DLCR Cohorts

#### 3.1.1. Baseline Characteristics

Comparing baseline characteristics between the DLCR pre-approval (*n* = 1658) and post-approval (*n* = 2055) cohorts showed a significant increase in the median age (from 68 to 70 years, *p* < 0.0001) (Table S1). Compared to the pre-approval cohort, the post-approval cohort comprised a significantly higher proportion of female patients (50.2% vs. 46.9%, *p* = 0.05) and adenocarcinomas (58.8% vs. 53.3%, *p* < 0.0001) (Table S1). Additionally, significant differences in TNM stage was found (*p* < 0.0001) before and after the implementation of ICIs; however, large differences in missing values were also observed (the post-approval cohort *n* = 246, the pre-approval cohort, *n* = 69) (Table S1). No differences in CCIS were found (Table S1).

The DLCR-ICI cohort (*n* = 482) had a larger proportion of female patients than the DLCR-CTx cohort (*n* = 1573) (58.3% vs. 47.7%, *p* < 0.0001) (Table S2). Significant differences were found in the distribution of NSCLC histopathology, with a higher proportion of squamous cell carcinomas in the DLCR-CTx cohort, and higher proportions of adenocarcinomas and “other” in the DLCR-ICI cohort (Table S2).

#### 3.1.2. OS before and after the Implementation of ICIs

**Figure 2:**
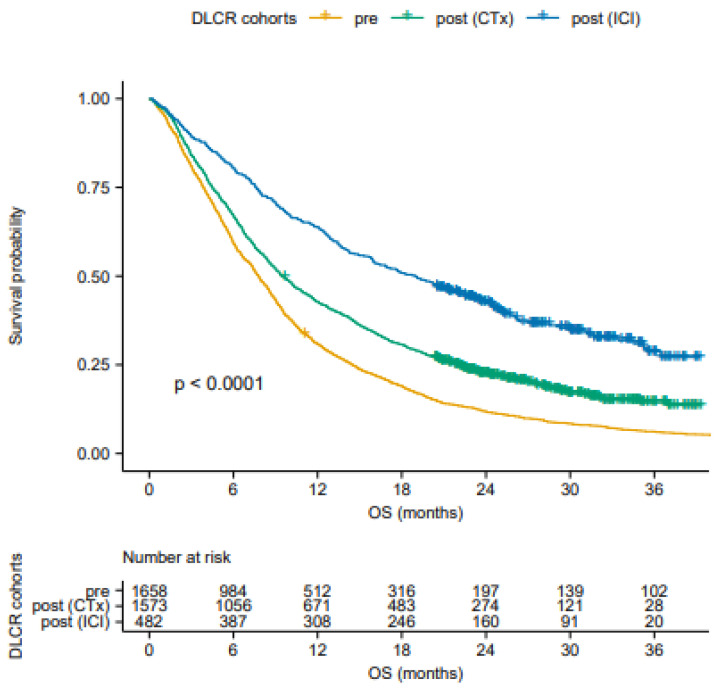
Overall survival (OS) of patients in Denmark before and after the approval of first-line immune checkpoint inhibitor (ICI). The survival of patients treated with chemotherapy (CTx) before the approval (pre) was compared to survival of patients treated with either CTx or ICI after the approval (post (CTx) and post (ICI)). DLCR, Danish Lung Cancer Registry.

Significant differences were seen in OS between the DLCR cohorts (*p*-value < 0.0001), with notable differences in mOS, and 1-, 2-, and 3-year survival rates (Figure 2 and Table 1). The greatest survival improvement was observed in patients receiving ICIs with a mOS increase from 7.8 months (95% CI 7.4–8.2) to 19.0 months (95% CI 16.0–22.0), 1-year OS rate from 31% to 64%, 2-year OS rate from 12% to 42% and 3-year OS rate from 6% to 29%.

### 3.2. The EHR-Identified ICI Cohort

#### 3.2.1. ICI Efficacy

The baseline characteristics of the EHR-identified ICI-treated patients (*n* = 579) are presented in Table 2.

ICI was administered following the primary diagnosis in 477 (82%) patients. The remaining patients received ICI after curatively intended surgery +/− adjuvant CTx (*n* = 39; 7%), chemoradiotherapy (CRT) (*n* = 46; 8%), or both (*n* = 16; 3%). PD-L1 was unknown or <50% in 27 patients (4.7%). The treatment data and reasons for treatment discontinuation are shown in Table 3.

At the censoring date, 38 patients (7%) were still on ICI treatment. The median follow-up period was 27.2 months (95% CI 26.7–28.2), and the median TTD was 4.8 months (95% CI 4.1–5.5) (Table S3).

PD was the most common reason for ICI discontinuation (*n* = 250, 46%), and half of the patients discontinued ICIs within six cycles (Figure S2). More reasons for ICI discontinuation were irAEs only (28%), poor PS (11%), completion of 2 years ICI (7%), and “other reasons” (9%) (Table 3). Following ICI treatment, systemic antineoplastic treatment was administered to 179 patients (33%). Of these patients, 28% received ≥ 2 treatment lines.

#### 3.2.2. Clinical Outcomes

The mOS was 18.3 months (95% CI 16.0–21.3); 15.2 (95% CI 13.0–18.3) in male and 21.5 (95% CI 18.0–25.1) in female patients. The mOS for patients with PS 0 was 28 months (95% CI 21.5–NR) compared to the 14.6 (95% CI 12.7–19.0) and 12.8 months (95% CI 7.6–16.1) in patients with PS 1 and PS ≥ 2, respectively. In patients with BoM, the mOS was 12.0 months (95% CI 9.5–14.9) compared to the 21.5 months (95% CI 19.0–24.9) in patients without. The mPFS was 8.2 months (95% CI 7.2–9.3); 7.1 (95% CI 6.0–8.5) in male and 8.8 (95% CI 7.9–11.8) in female patients. The mPFS for patients with PS 0 was 11.0 months (95% CI 8.5–13.9) compared to the 7.7 (95% CI 6.4–8.8) and 6.0 (95% CI 3.3–8.7) in patients with PS 1 and PS ≥ 2, respectively. In patients with BoM, the mPFS was 5.7 months (95% CI 4.4–7.8) compared to the 9.4 months (95% CI 8.1–12.0) in patients without.

For information on mOS and mPFS according to all baseline characteristics see Table S5.

In patients with PS 0–1, the estimated 3-year OS rate was 33% (95% CI 28–39) compared to the 25% (95% CI 16–39) in patients with PS ≥ 2. Furthermore, the mTTD for patients with PS ≥ 2 was 2.8 months (95% CI 1.4–4.2) (Table S3).

#### 3.2.3. Prognostic Clinical Factors

**Figure 3:**
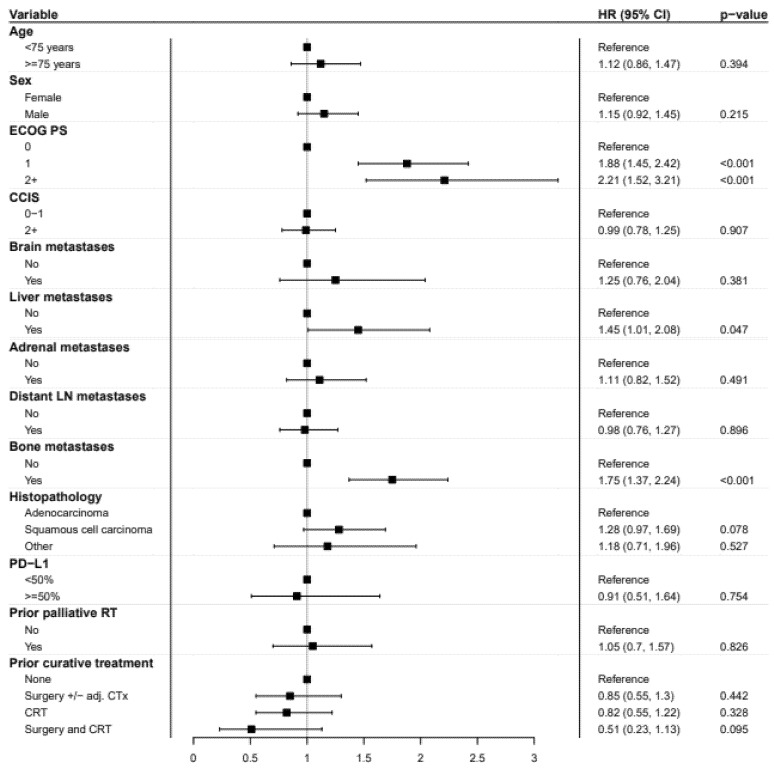
Weighted multivariable Cox regression analysis with forest plots showing average hazard ratios (HR) according to baseline characteristics. ECOG PS, European Cooperative Oncology Group performance status; CCIS, Charlson Comorbidity Index Score; RT, radiotherapy; CTx, chemotherapy; CRT, chemoradiotherapy.

KM estimates and log-rank tests showed that the OS was significantly reduced in male patients and in patients with PS ≥ 1, BoM, and/or liver metastases, and in patients who had received prior palliative RT (Table S4 and Figure S3). Baseline metastases in the brain, adrenal glands, and/or distant lymph nodes, age ≥ 75 years, CCIS ≥ 2, or prior curative treatment for NSCLC did not significantly affect OS (Table S5 and Figure S3). In the multivariable Cox regression analysis, PS 1 (HR = 1.86; 95% CI 1.44–2.39; *p* < 0.001) and PS ≥ 2 (HR = 2.19; 95% CI 1.5–3.18; *p* < 0.001), relative to PS 0, BoM (HR = 1.75; 95% CI 1.36–2.23; *p* < 0.001), and liver metastases (HR = 1.44; 95% CI 1.0–2.07; *p* = 0.05) remained independent of poor prognostic factors (Figure 3). Compared to patients with primary metastatic disease, patients with a relapse after prior curative treatment (surgery ± adjuvant CTx, curative CRT, or surgery + CRT) did not have a significantly improved OS.

In the interaction analysis of sex and histopathology, male patients with squamous cell carcinoma had significantly poorer survival than those with adenocarcinoma (HR = 1.70; 95% CI 1.18–2.47; *p* = 0.01). Univariable Cox regression results are given in Table S5.

## 4. Discussion

This nationwide Danish study was based on a consecutive cohort and demonstrated a significantly improved 3-year OS rate of 29% in 1L ICI-treated NSCLC patients compared to the 6% in those treated with 1L CTx before ICI implementation. However, more patients with PS ≥ 2 may have been treated with 1L CTx than 1L ICI as the Danish ICI recommendation applies to patients with PS 0–1 only. To our knowledge, this is the first RWS of patients with NSCLC without EGFR/ALK molecular alterations that included both large ICI cohorts and comparative cohorts since ICI treatment was implemented. An increase in OS in CTx-treated patients was also observed, possibly due to subsequent ICI treatment, earlier diagnosis (including potential lead time bias), stage migration owing to improved staging diagnostics, improved palliative care, changes in histopathological subtypes, advances in molecular testing, and sex distribution over time [26,27]. Those with PS ≥ 2 accounted for 15% of the ICI cohort in our RWS; however, these patients were not included in previous RCTs. This may partly explain the lower 3-year OS rate and mOS compared to those obtained in the KEYNOTE-024 and KEYNOTE-042 trials [6,7,8]. Furthermore, the poor OS of ICI-treated PS 1 patients in our study, could reflect a possible misclassification of PS 2 patients as PS 1 patients because 1L ICI was approved only for patients with PS 0-1. This issue complicates the comparison of PS data with other studies; however, this potential bias is not addressed in other RWS. In contrast, the mOS of PS 0 patients in our study was 28 months, comparable to that of patients in the KEYNOTE-024 study [6,7]. In line with other ICI RWS, we found PS ≥ 2 and liver metastases to be poor prognostic factors for OS [15,16,17,18,28]. Generally, the population of patients with PS 2 is heterogeneous and has worse clinical conditions due to comorbidities, higher tumor burden, or both [28,29]. Patients with BoM accounted for 28% in our study and had significantly worse mOS compared to patients without BoM. BoM has not been reported in RCTs and is rarely reported in other RWS [17,18]. However, this information is essential because the immune and skeletal systems are closely linked; for example, the receptor activator of nuclear factor-κB ligand (RANKL) stimulation suppresses T-cell killing and enhances immunosuppression in the bone tumor microenvironment [30,31]. Unfortunately, our RWS did not include information on the administration of bone-modifying agents. Clinical studies of the RANKL-inhibitor, denosumab, combined with ICIs are ongoing [32,33]. In our study, prior curative treatment did not significantly affect OS. However, tumor burden and the site of metastases at relapse, as well as the treatment strategy for oligometastatic relapse could affect the OS in these patients.

The majority of patients in our study were female (58%), as opposed to other RCTs and RWS, which reflects the higher proportion of female smokers in Denmark compared to that in other countries [34,35]. Furthermore, the proportion of female patients with NSCLC increased during the observed period.

A significant challenge with antineoplastic treatment (including ICIs) may be primary tumor resistance to treatment. In our study, 22% of patients experienced PD within six ICI cycles (i.e., 4.2 months of treatment). Various factors such as different PD-L1 intervals, inter- and intra-tumoral PD-L1 heterogeneity, host-immune-related mechanisms, and unidentified mutations such as STK11, along with currently unknown factors are possible explanations for early PD [36,37,38]. Those patients could potentially derive benefit from other 1L treatment options. Furthermore, pseudoprogression could be misinterpreted as PD in some cases. To optimize response evaluation in ICI-treated patients, the use of immune (i) RECIST could be implemented as a standard in the real-world setting as well as in the RCTs [39]. Additionally, a standardization of response evaluation could improve the comparability of ICI efficacy in RWS and RCTs.

RWS provide information on effectiveness in everyday clinical practice as they include patient subgroups not reported or included in RCTs [9,11]. Furthermore, new hypotheses can be generated from the RWS results. A major strength of this study is the substantial nationwide cohort, which provides new information on large consecutive subgroups seen in daily clinical practice, such as patients with PS ≥ 2, moderate-to-severe comorbidity, organ metastases, and age > 75 years. Furthermore, in the Danish Healthcare System, all patients have equal and free access to therapy, including ICIs (within the framework of national guidelines), thus lowering the risk of selection bias. The limitations of our study, and particularly related to the CTx-cohorts, are similar to those of other RWS with a retrospective design, which is the lack of data completeness and data accuracy.

Based on our results, some main questions still need to be answered to optimize the future ICI treatment of patients with advanced NSCLC. Primary resistance mechanisms in patients with early PD need to be further explored. In future RCTs, a higher representation of patients from daily clinical practice, and information on known prognostic factors such as metastatic load and location, is warranted. Prospective ICI investigations should focus on: differences between RCTs and routine care; complementary tools to assess patients’ daily living activities, frailty, and reasons leading to poor PS; possible differences between male and female patients. Furthermore, the optimal registration and research use of real-time clinical, molecular, and patient-reported data need to be established.

## 5. Conclusions

In this comprehensive nationwide study, we demonstrated that both the mOS and the long-term survival of real-world patients with advanced EGFR- and ALK negative NSCLC, treated with systemic antineoplastic treatment, has improved since the implementation of ICIs in Denmark. The survival of ICI-treated patients was lower than demonstrated in the RCTs, except for PS 0 patients. More than every fifth patient showed early PD within six cycles of ICI, and this group of patients especially may benefit from alternative treatments, if they could be identified upfront. PS ≥ 1, and bone and liver metastases were found to be significantly associated with worse mOS. Sex, CCIS, and age ≥ 75 years did not significantly affect the mOS.

## Figures and Tables

**Table 1:** Survival of patients with advanced NSCLC treated with systemic antineoplastic treatment before and after the introduction of ICIs.

DLCR Cohorts	*n* (%)	mOS (Months) (95% CI)	1-Year OS (%) (95% CI)	2-Year OS (%) (95% CI)	3-Year OS (%) (95% CI)
Pre-approval cohort	1658 (100)	7.8 (7.4–8.2)	31 (29–33)	12 (10–14)	6 (5–7)
Post-approval cohort	2055 (100)	11.0 (10.2–11.9)	48 (46–50)	27 (25–29)	18 (16–20)
CTx	1573 (77)	9.5 (8.9–10.3)	43 (40–45)	22 (21–25)	14 (12–17)
ICI	482 (23)	19.0 (16.0–22.0)	64 (60–68)	42 (38–47)	29 (24–35)

Median overall survival (mOS), 1-, 2-, and 3-year overall survival (OS) rates with 95% confidence interval (CI) before and after the approval of ICI treatment (the pre-approval cohort 1 January 2013–1 August 2014 and the post-approval cohort 1 March 2017–1 October 2018). NSCLC; non-small cell lung cancer; DLCR, Danish Lung Cancer Registry; *n*, number of patients; CTx, chemotherapy; ICI, immune checkpoint inhibitor.

**Table 2:** Baseline characteristics, ICI cohort.

Baseline Characteristics	*n* (%)
All patients	579
Age, median years (range)	70 (45–88)
<75	441 (76)
≥75	138 (24)
Sex	
Male	246 (42)
Female	333 (58)
ECOG performance status	
0	194 (34)
1	295 (51)
≥2	90 (15)
CCIS	
0 (none)	217 (37)
1 (mild)	169 (29)
2 (moderate)	103 (18)
3+ (severe)	90 (16)
Smoking status	
Current	189 (33)
Former	343 (59)
Never	26 (4)
Unknown	21 (4)
TNM stage and metastatic sites	
III	109 (19)
IV ^a^	470 (81)
Brain	38 (7)
Bone	162 (28)
Liver	63 (11)
Adrenal	86 (15)
Distant lymph nodes	174 (30)
NSCLC histopathology	
Adenocarcinoma	409 (71)
Squamous cell carcinoma	135 (23)
Other ^b^	35 (6)
PD-L1	
Negative ≥1% and <50%	3 (0.5) 20 (3.5)
≥50%	552 (95.3)
Unknown	4 (0.7)
Prior treatment with curative intention	
Surgery ± adj. CTx	39 (7)
CRT	46 (8)
Surgery and CRT	16 (3)
None	478 (82)
Prior palliative RT ^c^	
Yes	71 (12)
No	508 (88)

^a^ Patients may be registered with more than one metastatic site; ^b^ ‘Other’ includes NSCLC NOS (not otherwise specified) and adenosquamous carcinoma; ^c^ Prior palliative radiotherapy for NSCLC (primary lesion or metastatic site). *n*, number of patients; ECOG, Eastern Cooperative Oncology Group; CCIS, Charlson Comorbidity Index Score; TNM, tumor-node-metastasis classification of malignant tumors; NSCLC, non-small cell lung cancer; PD-L1, programmed death-ligand 1; adj. CTx, adjuvant chemotherapy; CRT, chemoradiotherapy; RT, radiotherapy.

**Table 3:** ICI treatment and irAEs.

Treatment Characteristics	*n* (%)
All patients	579
Median number of cycles (range)	7 (1–41)
Median days on treatment ^a^ (range)	127 (1–826)
Ongoing ICI treatment ^b^	38 (7)
ICI discontinuation	541 (93)
ICI discontinuation due to ^c^:	
PD	250 (46)
Poor performance status	62 (11)
Two years of ICI ^d^	39 (7)
IrAEs ^e^	170 (31)
Pneumonitis	41 (8)
Hepatitis	31 (6)
Skin	10 (2)
Endocrinopathy	18 (3)
Diarrhea/colitis	37 (7)
Other ^f^	52 (10)
IrAE only ^g^	150 (28)
Other reasons	51 (9)
Hospitalization due to irAE	135 (23)
Grade 5 toxicity (death)	12 (2)

^a^ Median time of ICI treatment = time to treatment discontinuation (TTD). ^b^ At date of censoring. ^c^ Each patient could be registered with more than one cause of treatment discontinuation. ^d^ Patients who received at least 2 years of ICI treatment. ^e^ Each patient could be registered with more than one type of irAE as a cause of treatment discontinuation. Percentage (in parentheses) describes the proportion of patients who stopped ICI because of the specific irAE compared to all patients who discontinued ICI (*n* = 541) ^f^ “Other” are not specified irAEs. ^g^ Proportion of patients with irAE as the only cause of treatment discontinuation. ICI, immune checkpoint inhibitor; irAE, immune-related adverse event; *n*, number of patients; PD, progressive disease.

## Data Availability

The study data can be available on request from the corresponding author, Mette T Mouritzen. The data are not publicly available due to the General Data Protection Regulation.

## Supplementary Materials

The following are available online at https://www.mdpi.com/article/10.3390/cancers13194846/s1, Figure S1: Criteria applied to the DLCR dataset, Figure S2: ICI treatment discontinuation due to progressive disease, Figure S3: Kaplan–Meier curves for the EHR-identified ICI cohort according to age, bone metastases, performance status, and sex and histopathology, Table S1: Comparison of baseline characteristics in the DLCR pre- and post-approval cohorts, Table S2: Comparison of baseline characteristics in the post-approval DLCR-CTx and DLCR-ICI cohorts, Table S3: Time to treatment discontinuation (TTD), Table S4: Median OS and PFS according to selected baseline characteristics of ICI-treated patients, Table S5: Univariable Cox regression analysis.
