# Supplementary material for: Nationwide Survival Benefit after Implementation of First-Line Immunotherapy for Patients with Advanced NSCLC—Real World Efficacy
Source: Cancers (Basel). 2021 Sep 28;13(19):4846. doi: 10.3390/cancers13194846 (PMC8507718; doi:10.3390/cancers13194846)
Supplement: Supplementary file 1 [file cancers-13-04846-s001.zip › cancers-1377287-supplementary.pdf]

Supplementary Materials: Nationwide Survival Benefit after Implementation of First-Line Immunotherapy for Patients with Advanced NSCLC—Real World Efficacy

Mette T. Mouritzen, Andreas Carus, Morten Ladekarl, Peter Meldgaard, Anders Winther Mølby Nielsen, Anna Livbjerg, Jacob W. Larsen, Halla Skuladottir, Charlotte Kristiansen, Kim Wedervang, Tine Schytte, Karin H. Hansen, Anne-Cathrine Østby, Malene S. Frank, Jakob Lauritsen, Jens B. Sørensen, Seppo W. Langer, Gitte F. Persson, Jon L. Andersen, Johanna M. C. Frary, Lars B. Drivsholm, Charles Vesteghem, Heidi S. Christensen, Birgitte Bjørnhart and Mette Pøhl

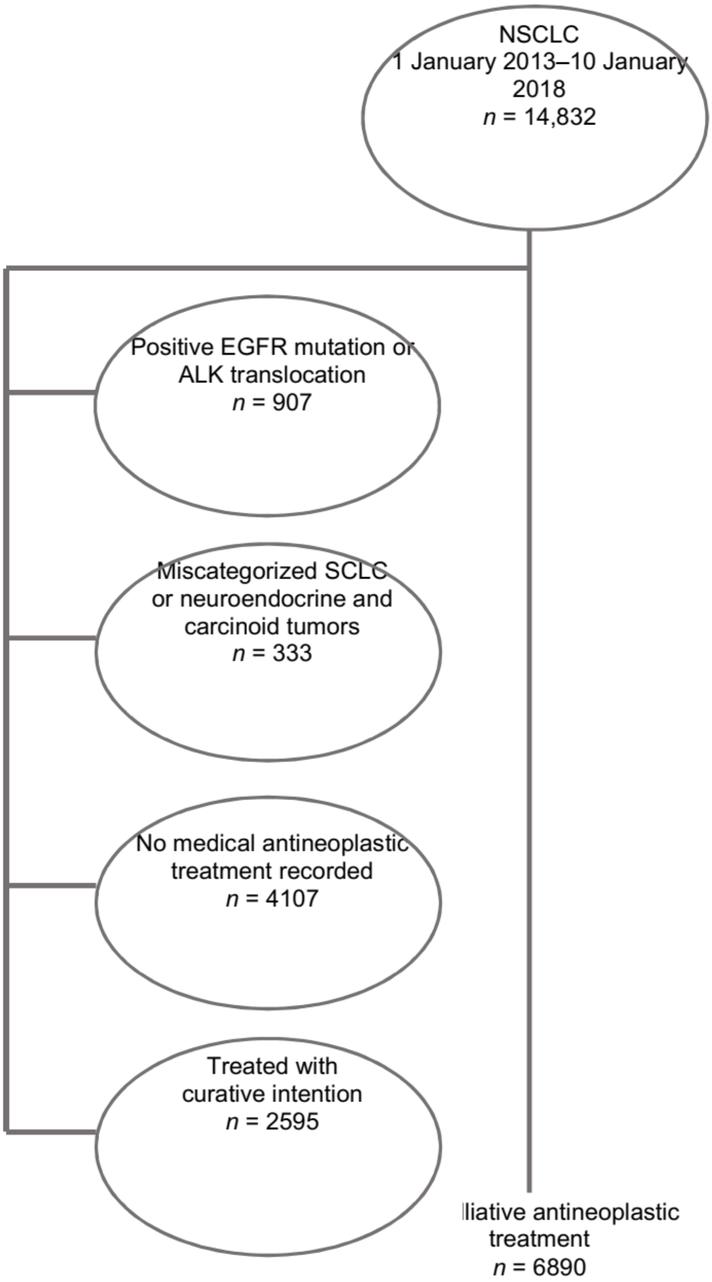

Figure S1. Criteria applied to the DLCR dataset.

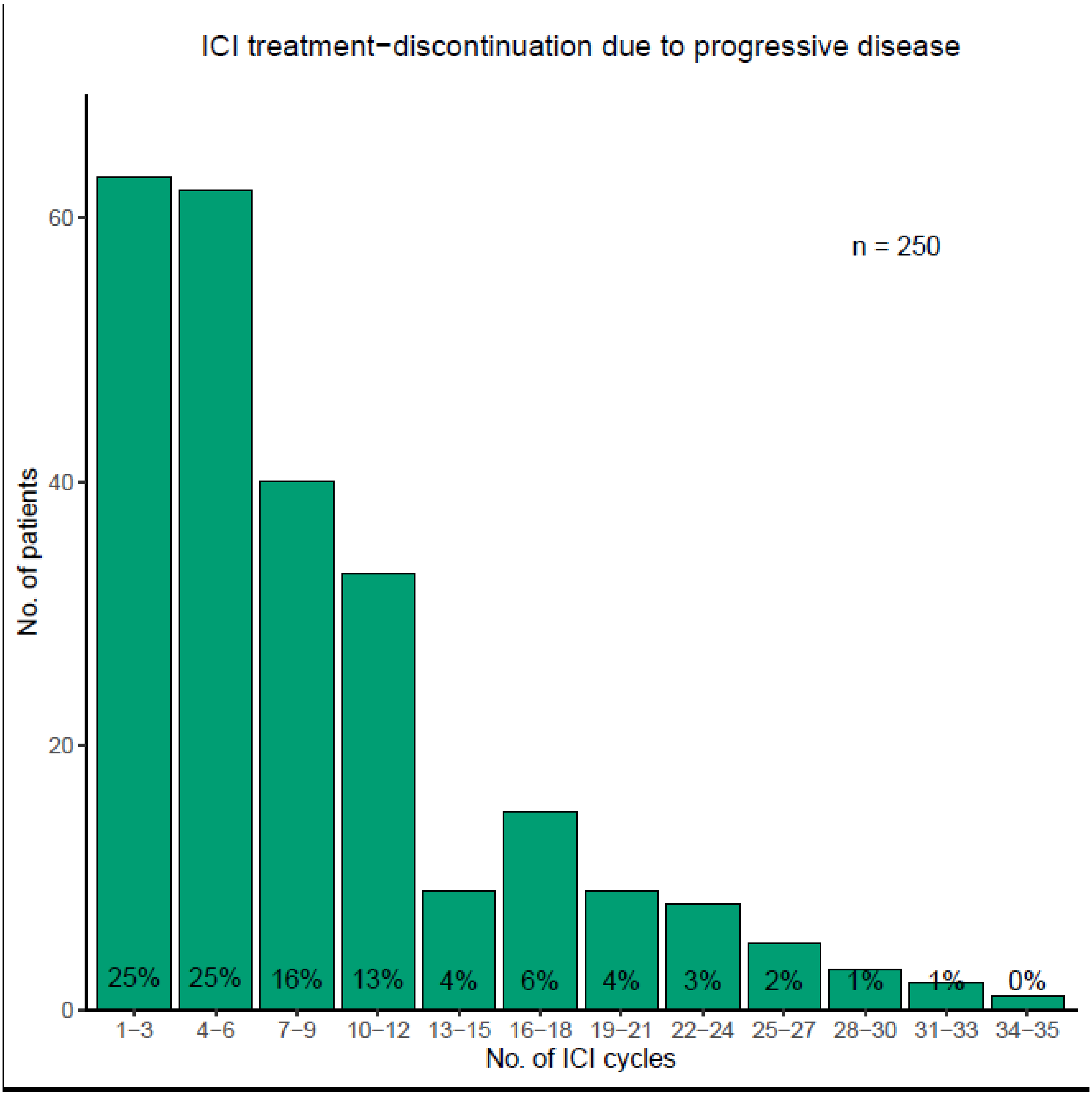

Figure S2. ICI treatment discontinuation due to progressive disease.

Figure S2:

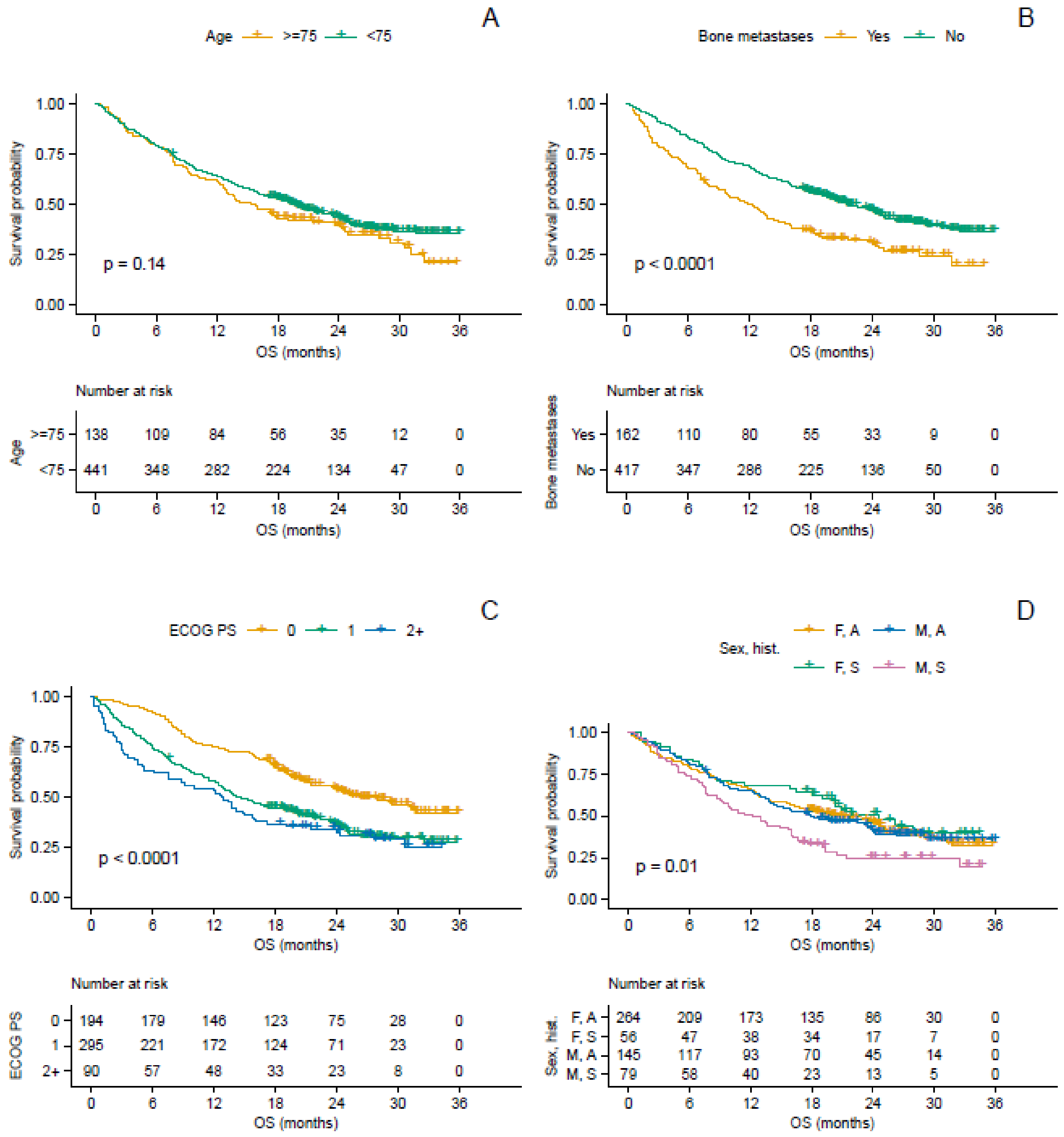

Figure S3. Kaplan–Meier curves for the EHR-identified ICI cohort according to age, bone metastases, performance status, and sex and histopathology.

Table S1: Comparison of baseline characteristics in the DLCR pre- and post-approval cohorts

**Baseline Characteristics**	**DLCR Pre-Approval Cohort *n* (%)**	**DLCR Post-Approval Cohort *n* (%)**	***p*-Value**
All patients	1,658	2,055	
Age, median years (range)	68 (31–91)	70 (32–91)	<0.0001
Age			
≥75 years	361 (21.8)	495 (24.1)	0.10
<75 years	1,297 (78.2)	1,560 (75.9)	
Sex			
Male	880 (53.1)	1,023 (49.8)	0.05
Female	778 (46.9)	1,032 (50.2)	
CCIS			
0	859 (51.8)	1,109 (54.0)	0.42
1	356 (21.5)	424 (20.6)	
2+	443 (26.7)	522 (25.4)	
TNM stage[a]			
IIIa	113 (7.1)	254 (14.0)	
IIIb	196 (12.3)	261 (14.4)	
IV	1,194 (75.1)	1,223 (67.6)	<0.0001
None	86 (5.4)	71 (3.9)	
Missing	69	246	
NSCLC histopathology			
Adenocarcinoma	884 (53.3)	1,209 (58.8)	
Squamous cell carcinoma	329 (19.8)	473 (23.0)	<0.0001
Other	445 (26.8)	373 (18.2)	

Comparison of baseline characteristics in the DLCR pre- and post-approval cohorts. a) TNM classification of malignant tumors, 7th edition. DLCR, Danish Lung Cancer Registry; *n*, number; CTx, chemotherapy; ICI, immune checkpoint inhibitor; n, number of patients; CCIS, Charlson Comorbidity Index Score; TNM, tumor-node-metastasis classification of malignant tumors; NSCLC, non-small cell lung cancer.

Table S2. Comparison of baseline characteristics in the post-approval DLCR-CTx and DLCR-ICI cohorts.

**Baseline Characteristics**	**DLCR-CTx *n* (%)**	**DLCR-ICI *n* (%)**	***p*-Value**
All patients	1,573	482	
Age, median years (range)	70 (32–91)	70 (45–88)	0.98
Age			
≥75 years	373 (23.7)	122 (25.3)	0.51
<75 years	1,200 (76.3)	360 (74.7)	
Sex			
Male	822 (52.3)	201 (41.7)	<0.0001
Female	751 (47.7)	281 (58.3)	
CCIS			
0	831 (52.8)	278 (57.7)	0.15
1	329 (20.9)	95 (19.7)	
2+	413 (26.3)	109 (22.6)	
TNM stage[a]			
IIIa	202 (14.8)	52 (11.8)	
IIIb	204 (14.9)	57 (12.9)	
IV	905 (66.2)	318 (71.9)	0.16
None	56 (4.1)	15 (3.4)	
Missing	206	40	
NSCLC histopathology		291 (60.4)	
Adenocarcinoma	918 (58.4)	92 (19.1)	0.04
Squamous cell carcinoma	381 (24.2)	99 (20.5)	

Other	274 (17.4)	

Comparison of baseline characteristics in the post-approval DLCR-CTx and DLCR-ICI cohorts. a) TNM classification of malignant tumors, 7th edition. DLCR, Danish Lung Cancer Registry; CTx, chemotherapy; ICI, immune checkpoint inhibitor; n, number of patients; CCIS, Charlson Comorbidity Index Score; TNM, tumor-node-metastasis classification of malignant tumors; NSCLC, non-small cell lung cancer.

Table S3. Time to treatment discontinuation (TTD).

**ICI Cohort**	***n* (%)**	**mTTD (Months) (95% CI)**
All patients	579 (100)	4.8 (4.1–5.5)
Age		
<75 years	441 (76)	4.9 (4.1–5.7)
≥75 years	138 (24)	4.0 (3.4–6.2)
Sex		
Male	246 (42)	4.7 (3.6–5.8)
Female	333 (58)	5.0 (3.8–5.9)
ECOG PS		
0	194 (34)	6.9 (5.1–9.7)
1	295 (51)	4.3 (3.5–5.5)
≥2	90 (16)	2.8 (1.4–4.2)
CCIS		
0–1	386 (67)	4.7 (3.6–5.6)
2+	193 (33)	5.4 (3.8–6.3)
TNM stage		
III	109	5.4 (4.6–7.8)
IV	470	4.5 (3.6–5.5)
Brain metastases		
Yes	38 (7)	4.5 (2.2–7.8)
No	541 (93)	4.8 (4.1–5.5)
Bone metastases		
Yes	162 (28)	3.5 (2.5–4.8)
No	417 (72)	5.5 (4.7–6.9)
Liver metastases		
Yes	63 (11)	3.5 (1.8–6.2)
No	516 (89)	4.9 (4.1–5.7)
Adrenal metastases		
Yes	86 (15)	5.1 (2.6–7.6)
No	493 (85)	4.8 (3.9–5.6)
Distant lymph node metastases		
Yes	174 (30)	5.5 (3.5–6.5)
No	405 (70)	4.7 (3.8–5.5)
NSCLC histopathology		
Adenocarcinoma	409 (71)	5.0 (4.1–5.8)
Squamous cell carcinoma	135 (23)	3.7 (3.5–5.6)
Other	35 (6)	4.7 (2.8–13.1)
PD-L1		
<50 %	23 (4)	5.5 (2.9–7.7)
≥50 %	552 (95)	4.8 (3.8–5.5)
Unknown	4 (1)	
Prior treatment with curative intention		
Surgery +/- adj. CTx	39 (7)	11.7 (8.7–18.7)
CRT	46 (8)	10.9 (8.2–20.3)
Surgery and CRT	16 (3)	19.1 (6.6–NR)
None	478 (83)	11.1 (9.1–12.7)
Prior palliative RT		
Yes	71 (12)	8.9 (7.2–13.8)
No	508 (88)	11.5 (9.7–12.8)

Time to treatment discontinuation (TTD) with 95% confidence intervals (CI) according to selected baseline characteristics. ICI, immune-checkpoint inhibitor; n, number of patients; mTTD, median time to treatment discontinuation; ECOG PS, Eastern Cooperative Oncology Group performance status; CCIS, Charlson Comorbidity Index Score; TNM, tumor-node-metastasis classification of malignant tumors; NSCLC, non-small cell lung cancer; PD-L1, programmed death-ligand 1; adj. CTx, adjuvant chemotherapy; CRT, chemoradiotherapy; RT, radiotherapy.

Table S4. Median OS and PFS according to selected baseline characteristics of ICI-treated patients.

ICI Cohort	*n* (%)	mOS (Months) (95% CI)	Log-Rank Test	mPFS (Months) (95% CI)	Log-Rank Test
All patients	579 (100)	18.3 (16.0–21.3)		8.2 (7.2–9.3)	
Age					
<75 years	441 (76)	19.6 (16.5–23.1)	0.13	8.2 (7.4–9.8)	0.29
≥75 years	138 (24)	15.6 (12.8–23.6)		7.9 (6.0–10.3)	
Sex					
Male	246 (42)	15.2 (13.0–18.3)	0.03	7.1 (6.0–8.5)	0.57
Female	333 (58)	21.5 (18.0–25.1)		8.8 (7.9–11.8)	
ECOG PS					
0	194 (34)	28.0 (21.5–NR)	< 0.0001	11.0 (8.5–13.9)	0.00
1	295 (51)	14.6 (12.7–19.0)		7.7 (6.4–8.8)	
≥2	90 (16)	12.8 (7.6–16.1)		6.0 (3.3–8.7)	
CCIS					
0–1	386 (67)	19.0 (15.9–23.1)	0.85	8,2 (6,9–9,9)	0.64
2+	193 (33)	17.2 (15.3–23.5)		8,0 (6,6–9,8)	
TNM stage					
III	109	20.2 (14.6–29.0)	0.39	8.8 (7.1–13.6)	0.11
IV	470	17.7 (15.8–21.4)		8.0 (6.8–9.2)	
Brain metastases					
Yes	38 (7)	17.1 (8.2–24.1)	0.16	11.3 (3.7–12.8)	0.20
No	541 (93)	19.0 (16.0–21.7)		8.1 (7.1–8.9)	
Bone metastases					
Yes	162 (28)	12.0 (9.5–14.9)	< 0.0001	5.7 (4.4–7.8)	< 0.0001
No	417 (72)	21.5 (19.0–24.9)		9.4 (8.1–12.0)	
Liver metastases					
Yes	63 (11)	13.4 (6.0–21.4)	0.00	3.9 (2.5–12.8)	0.06
No	516 (89)	19.0 (16.1–22.5)		8.3 (7.6–9.5)	
Adrenal metastases					
Yes	86 (15)	15.8 (12.5–24.1)	0.23	8.3 (5.4–12.4)	0.22
No	493 (85)	19.0 (16.4–21.5)		8.1 (7.1–9.4)	
Distant lymph node metastases					
Yes	174 (30)	19.6 (15.7–25.0)	0.65	8.7 (6.9–12.3)	0.18
No	405 (70)	17.6 (15.3–20.5)		8.0 (6.5–9.2)	
NSCLC histopathology					
Adenocarcinoma	409 (71)	19.6 (16.4–24.0)	0.33	8.5 (7.8–10.7)	0.13
Squamous cell carcinoma	135 (23)	16.0 (12.1–20.2)		6.7 (5.8–8.2)	
Other	35 (6)	19.1 (9.33–NR)		12.0 (3.4–NR)	
PD–L1					
<50 %	23 (4)	14.9 (9.3–NR)	0.80	7.0 (5.0–12.0)	0.09
≥50 %	552 (95)	18.3 (16.0–21.5)		8.2 (7.3–9.4)	
Unknown	4 (1)				
Prior treatment with curative intention					
Surgery +/- adj. CTx	39 (7)	19.4 (13.8–NR)	0.72	9.2 (7.6–18.3)	0.76
CRT	46 (8)	18.3 (13.7–NR)		8.1 (7.0–13.0)	
Surgery and CRT	16 (3)	24.4 (9.8–NR)		12.3 (6.0–NR)	
None	478 (83)	18.0 (15.8–21.3)		8.0 (6.6–8.8)	
Prior palliative RT	71 (12)	13.8 (10.1–21.8)	0.03	7.4 (4.0–12.5)	0.23

Yes	508 (88)	19.0 (16.1–22.5)	8.2 (7.3–9.4)
No			

Median overall survival (mOS) and median progression-free survival (mPFS) with 95% confidence interval (CI) according to baseline characteristics. ICI, immune checkpoint inhibitor; *n*, number of patients; ECOG PS, Eastern Cooperative Oncology Group performance status; CCIS, Charlson Comorbidity Index Score; TNM, tumor-node-metastasis classification of malignant tumors; NSCLC, non-small cell lung cancer; PD-L1, programmed death-ligand 1; adj. CTx, adjuvant chemotherapy; CRT, chemoradiotherapy; RT, radiotherapy; NR, not reached.

Table S5. Univariable Cox regression analysis.

ICI Cohort	*n* (%)	HR (95% CI)	*p*-Value
Age			
<75 years	441 (76)	Reference	
≥75 years	138 (24)	1.23 (0.97–1.56)	0.09
Sex			
Female	333 (58)	Reference	
Male	246 (42)	1.22 (0.99–1.52)	0.07
ECOG PS			
0	194 (34)	Reference	
1	295 (51)	1.80 (1.42–2.29)	< 0.001
≥2	90 (16)	2.28 (1.63–3.2)	< 0.001
CCIS			
0-1	386 (67)	Reference	
2+	193 (33)	0.99 (0.79–1.24)	0.91
Brain metastases			
No	541 (93)	Reference	
Yes	38 (7)	19.0 (0.87–1.98)	0.19
Bone metastases			
No	417 (72)	Reference	
Yes	162 (28)	1.77 (1.40–2.23)	< 0.001
Liver metastases			
No	516 (89)	Reference	
Yes	63 (11)	1.73 (1.24–2.42)	0.00
Adrenal metastases			
No	493 (85)	Reference	
Yes	86 (15)	1.24 (0.93–1.66)	0.14
Distant lymph node metastases			
No	405 (70)	Reference	
Yes	174 (30)	1.00 (0.79–1.27)	0.99
NSCLC histopathology			
Adenocarcinoma	409 (71)	Reference	
Squamous cell carcinoma	135 (23)	1.18 (0.92–1.52)	0.18
Other	35 (6)	1.12 (0.69–1.78)	0.67
PD-L1			
<50 %	23 (4)	Reference	
≥50 %	552 (95)	0.98 (0.57–1.69)	0.94
Prior treatment with curative intention			
None	478 (83)	Reference	0.67
Surgery +/- adj. CTx	39 (7)	0.91 (0.59–1.41)	0.23
CRT	46 (8)	0.80 (0.56–1.15)	0.30
Surgery and CRT	16 (3)	0.69 (0.34–1.39)	
Prior palliative RT			
No	508 (88)	Reference	
Yes	71 (12)	1.42 (1.03–1.96)	0.03

Weighted univariable Cox regression analysis with hazard ratios (HRs) according to baseline characteristics. PS 1, PS ≥2, liver metastases, bone metastases and prior palliative radiotherapy (RT) are associated with significantly reduced survival. ICI, immune checkpoint inhibitor; *n*, number of patients; ECOG PS, Eastern Cooperative Oncology Group performance

status; CCIS, Charlson Comorbidity Index Score; NSCLC, Non-small cell lung cancer; PD-L1, programmed death-ligand 1; adj. CTx, adjuvant chemotherapy; CRT, chemoradiotherapy; RT, radiotherapy.
